# Soluble biomarkers of HIV-1-related systemic immune activation are associated with high plasma levels of growth factors implicated in the pathogenesis of Kaposi sarcoma in adults

**DOI:** 10.3389/fimmu.2023.1216480

**Published:** 2023-09-18

**Authors:** Benderli Christine Nana, Livo Forgu Esemu, Michael Ebangha Besong, Derrick Hyacinthe Nyasse Atchombat, Kazuhiro Ogai, Thérèse M. Patricia Sobgui, Chris Marco Mbianda Nana, Reine Medouen Ndeumou Seumko’o, Honoré Awanakan, Gabriel Loni Ekali, Rose Gana Fomban Leke, Shigefumi Okamoto, Lishomwa C. Ndhlovu, Rosette Megnekou

**Affiliations:** ^1^ The Immunology Laboratory of the Biotechnology Center, University of Yaoundé I, Yaounde, Cameroon; ^2^ Department of Animals Biology and Physiology of the Faculty of Sciences, University of Yaoundé I, Yaoundé, Cameroon; ^3^ Department of Biomedical Sciences of the Faculty of Health Sciences, University of Buea, Buea, Cameroon; ^4^ Centre for Research on Emerging and Reemerging Diseases, Institute of Medical Research and Medicinal Plant Studies, Yaounde, Cameroon; ^5^ Faculty of Health Sciences, Institute of Medical, Pharmaceutical and Health Sciences, Kanazawa University, Kanazawa, Japan; ^6^ Division of Infectious Diseases, Department of Medicine, Weill Cornell Medicine, New York, NY, United States

**Keywords:** HIV-1, systemic immune activation, IgG anti HHV8, VEGF, FGF acidic

## Abstract

**Background:**

Human Herpesvirus-8 (HHV-8) is the etiologic agent of Kaposi’s sarcoma (KS), a multicentric angio-proliferative cancer commonly associated with Human Immunodeficiency Virus (HIV) infection. KS pathogenesis is a multifactorial condition hinged on immune dysfunction yet the mechanisms underlying the risk of developing KS in HHV-8 seropositive adults remains unclear. Here we explored whether soluble markers of HIV-1-related systemic immune activation (SIA) and angiogenesis (VEGF and FGF acidic) are involved in the pathogenesis of KS in adults with HHV8.

**Methodology:**

Blood samples from 99 HIV-1 infected and 60 HIV-1 uninfected adults were collected in Yaoundé, Cameroon. CD3+/CD4+ T cell counts and HIV-1 plasma viral load were determined using the Pima Analyzer and the RT-PCR technique, respectively. Plasma levels of SIA biomarkers (sCD163, sCD25/IL-2Rα, and sCD40/TNFRSF5) and biomarkers of progression to KS (VEGF and FGF acidic) were measured using the Luminex assay. Seropositivity (IgG) for HHV-8 was determined using the ELISA method.

**Results:**

Overall, 20.2% (20/99) of HIV-1 infected and 20% (12/60) of HIV-1 uninfected participants were seropositive for HHV8. Levels of sCD163, sCD25/IL-2Rα, sCD40/TNFRSF5, and FGF acidic were higher in the HIV-1 and HHV8 co-infection groups compared to the HIV-1 and HHV8 uninfected groups (all P <0.05). In addition, Higher plasma levels of VEGF correlated with sCD163 (r_s_ = 0.58, P =0.0067) and sCD40/TNFRSF5 (r_s_ = 0.59, P = 0.0064), while FGF acidic levels correlated with sCD40/TNFRSF5 (r_s_ = 0.51, P = 0.022) in co-infected. In HIV-1 mono-infected donors, VEGF and FGF acidic levels correlated with sCD163 (r_s_ =0.25, P = 0.03 and r_s_ = 0.30, P = 0.006 respectively), sCD25/IL-2Rα (r_s_ = 0.5, P <0.0001 and r_s_ = 0.55, P <0.0001 respectively) and sCD40/TNFRSF5 (r_s_ = 0.7, P <0.0001 and r_s_ = 0.59, P <0.0001 respectively) and even in patients that were virally suppressed sCD25/IL-2Rα (r_s_ = 0.39, P = 0.012 and r_s_ = 0.53, P = 0.0004 respectively) and sCD40/TNFRSF5 (r_s_ = 0.81, P <0.0001 and r_s_ = 0.44, P = 0.0045 respectively).

**Conclusion:**

Our findings suggest that although the development of KS in PLWH is multifactorial, HIV-associated SIA might be among the key drivers in coinfections with HHV8 and is independent of the patients’ viremic status.

## Introduction

Human Herpesvirus-8 (HHV-8) or Kaposi’s sarcoma-associated herpesvirus (KSHV) is the etiologic agent of Kaposi’s sarcoma (KS), the most common HIV/AIDS-associated cancer (1–3). Until the advent of the AIDS epidemic, KS was found in specific populations, notably individuals from equatorial Africa (endemic KS), older men from Eastern Europe and the Mediterranean (classic KS), and individuals who received an organ transplant or were immune compromised (iatrogenic KS) (2). Despite being a relatively infrequent malignancy worldwide, KS remains the most common cancer in People Living with HIV (PLWH) in the African continent.

Although the widespread use of antiretroviral therapy (ART) has led to a remarkable decline in KS incidence in PLWH (4), the risk of developing KS in PLWH remains substantially higher than in the general population (5). KS has a viral etiology and multifactorial pathogenesis hinged on immune dysfunction (6). HHV-8 infection can be considered a necessary but not a sufficient condition for the development of KS.

Persistent systemic immune activation (SIA) and inflammation is a hallmark of HIV infection and is strongly associated with AIDS and non-AIDS morbidity and mortality. Several studies demonstrated that plasma levels of immune activation and inflammation biomarkers, as well as those of their mediators, have improved with the introduction of ART (7), but the levels are still higher in PLWH compared to HIV-uninfected populations (8–11). T-cell activation (12), and monocyte/macrophage (13, 14) biomarkers are persistently higher among PLWH, and are implicated in the immunopathogenesis of a wide range of infections in PLWH (15, 16). It has been demonstrated that circulating levels of sCD40/TNFRSF5 and sCD25/IL-2R alpha, both lymphocyte activation biomarkers are elevated in patients with chronic renal failure, chronic liver diseases, and hematological malignancies (17–20) which also affect people with chronic HIV. But their role in other cancers such as KS, an angio-proliferative cancer commonly associated with HIV infection remains unclear.

Angiogenesis is a fundamental event in the process of tumor growth and metastatic dissemination. Angiogenic mediator like Vascular Endothelial Growth Factor (VEGF) has recently been postulated as a major angiogenic and growth factor in several cancer including KS (21). In synergy with Fibroblast Growth Factor (bFGF), VEGF induces endothelial cell growth and vascular permeability in HIV-associated KS (22–24). The question that arises is whether HIV-associated SIA potentially drives angiogenesis in the pathogenesis of KS in co-infections with HHV8? This study therefore aims to evaluate the modulation of soluble biomaker plasma levels of HIV-1-related SIA and that of growth factors (VEGF and FGF acidic) in HIV-1 and HHV8 co-infected adults, in order to define the relationships between these markers across infection status. This may help to preconize a better management and reduce opportunistic infection in adult living with HIV.

## Methods

### Study design and population

This cross-sectional study was carried out between June 2018 and July 2019 at the HIV clinic of the Efoulan District Hospital and Central Hospital in Yaoundé, Cameroon. These Hospitals were chosen due to an existing collaboration with the hospitals and University research center and of the high number of people living with HIV visiting these treatment centers. A total of 159 out-patients and in-patients aged 21 to 55 years were enrolled. Socio-demographic and clinical information was recorded. HIV-uninfected participants were defined by having negative HIV rapid diagnostic test (RDT) (Alere Determine HIV-1/2, Matsudo, Japan and Oraquick HIV-1/2, Orasure Technologies, Inc) while HIV-infected participants were defined by a positive HIV RDT result. Pregnant women and individuals with malaria infection were excluded from this study to minimize the effect of these conditions on the plasma concentration of biomarkers used in this study. In fact, we wanted to avoid biases due to malaria specific immune responses and pregnancy status, given the fact that pregnancy is characterised by physiological changes that can lead to immunosuppression. Peripheral blood samples were collected in EDTA tubes from all participants. A portion of the blood was used to quantify CD3+/CD4+ Lymphocyte T (LT) cell count and the leftover was centrifuged to collect plasma for storage at -80° C, viral load quantification, and the measurement of immune markers of interest.

### CD3+/CD4+ T cell count quantification

Freshly collected EDTA whole blood (25µL) was used to determine CD3+/CD4+ T cell counts using the Pima Analyser (Alere Technologies GmbH; Loebstedter Str. 103-105 D-07749 Jena; Germany), as described by the manufacturer. The whole blood samples were charged and inserted into the Pima CD4 test cartridge in the Analyzer, then transported in the incubation compartment where it interacted with the following specific antibodies: an anti-human CD3 monoclonal antibody conjugated to dye 1 and an anti-human CD4 monoclonal antibody conjugated to dye 2. Fluorescence signals were detected by an integrated camera and analyzed using the embedded proprietary software algorithms on the built-in computer. T-cell auxiliaries present both CD3 and CD4 surface antigens and emit a signal light at specific wavelengths to the two antibody-dye conjugates. The results were automatically generated by the Pima Analyzer and the cell counts were expressed per µL.

### HIV-1 serology determination and plasma viral load quantification by real-time PCR

**Figure 1:**
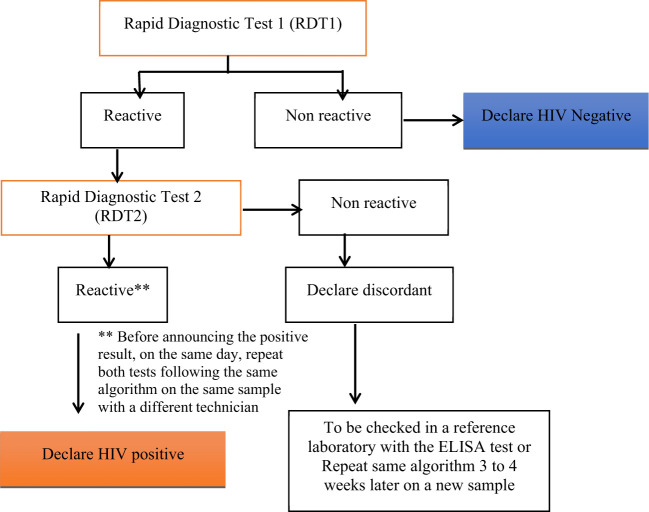
National algorithm for HIV Rapid Test in Cameroon, RDT1, highly sensitive (Alere Determine HIV-1/2, Matsudo, Japan); and RDT2, highly specific (Oraquick HIV-1/2, Orasure Technologies, Inc).

HIV-1 serology was determined according to the National algorithm for HIV Rapid Diagnosis Test (RDT) (25) in Cameroon as described in **Figure 1**. Whole blood samples were first tested with the RDT1, a highly sensitive test (Alere Determine HIV-1/2, Matsudo, Japan). If the test was reactive for RDT1, a highly specific test RDT2 (Oraquick HIV-1/2, Orasure Technologies, Inc) was used for the confirmation. When reactive with RDT2, the sample was considered positive for HIV but if the RDT2 was nonreactive, the sample was considered indeterminate and an ELISA test (AiD™ anti-HIV 1 + 2 ELISA, Beijing Wantai Biological Pharmacy Enterprise Co., Ltd) was performed to confirm the result. A sample was considered negative for HIV if the RDT1 was non-reactive. Viral load was determined as described by Livo et al. (26). Undetectable viral load threshold was set at <40 copies/mL.

To quantify HIV-1 plasma viral load, the Reverse Transcriptase-Polymerase Chain Reaction (RT-PCR) technique was performed using a RealTime HIV-1 kit on an Abbott m2000rt instrument (Abbott Molecular Inc. 1300 East Tauhy Avenue Des Plaines, IL 60018 USA), according to the manufacturer’s instructions. In brief, an RNA sequence that is unrelated to the HIV-1 target sequence was introduced into each sample at the beginning of sample preparation. The amount of HIV-1 target sequence that was present at each amplification cycle was measured through the use of fluorescent-labeled oligonucleotide probes on the Abbott m2000rt instrument. The amplification cycle at which fluorescent signal is detected by the Abbott m2000rt was proportional to the log of the HIV-1 RNA concentration present in the original sample.

### Serology test for HHV8

A Human herpes virus-8 IgG Antibody ELISA Kit was used to measure Herpes virus-8 IgG antibodies following the manufacturer’s protocols (MyBioSource.com, USA). Briefly, after adding positive control, negative control, and diluted samples in the corresponding well of the micro–ELISA Strip plate, HRP-conjugate Anti-Human IgG antibody was added, and the plates were incubated at 37°C for 60 minutes. After washing plates five times with washing buffer, the substrate was added, and the plate was incubated for 15 minutes at 37°C. Thereafter, the stop solution was added and the Optical Densities (O.D.) were read within 15 minutes at 450 nm using a microplate reader (Spectra Max plus microplate Spectrophotometer, Sunnyvale, CA94089, USA). The results were determined according to the protocol by calculating the critical CUT OFF (Critical CUT OFF= the average of Negative control well + 0.15). Results were considered positive if sample OD was ≥ calculated critical CUT-OFF OFF and negative if sample OD was < calculated critical CUT-OFF. This test provided qualitative, but not a quantitative determination of IgG antibodies.

### Measurement of soluble biomarkers of systemic immune activation and growth factors

Plasma levels of soluble biomarkers of SIA (sCD163, sCD25/IL-2R alpha, sCD40/TNFRSF5) and two growth factors (VEGF, FGF acidic) were measured by magnetic Luminex screening assay, using Human Premixed Multi-Analyte Kit (R&D Systems, Inc. Minneapolis, MN, USA). The assay was carried out following the protocol and instructions of the manufacturer. Briefly, plates were incubated with 50 µL/well of each diluted sample and different concentrations of standard cytokines and with 50 µL/well of Human Magnetic Premixed Microparticle Cocktail containing antibodies specific for each of the analytes. After washing with wash buffer three times using a magnetic plate separator (Luminex, Austin, TX, USA, Cat# CN-0269-01), plates were incubated with 50 µL/well of Human Premixed Biotin-antibodies cocktail specific for each of the analytes. The second washing step was performed, and antibody-analyte complexes were revealed using 50 µL/well of Streptavidin-PE. Plates were then incubated for 30 minutes and following the last washing step, 100 µL/well of wash buffer was added to resuspend the microparticles. Plates were read after 2 minutes of incubation using a Luminex MAGPix Analyzer (XMAP Technology, SN, USA). All the incubation steps were at room temperature on the horizontal shaker. Results were expressed as Median Fluorescence Intensity (MFI). A standard curve was generated for each analyte to convert MFI into corresponding analyte relative concentration.

### Statistical analyses

The GraphPad Prism 8.4.3 software was used for statistical analyses. Results were reported as medians with the interquartile range for continuous variables such as age, educational levels, LT CD4 count, and Duration on ARTs, or *n* (%) where appropriate. Mann–Whitney rank sum test was used to evaluate inter-group differences. Spearman rank order correlation (r_s_) was used to evaluate parameter association between the different groups. Proportions were compared using Fisher’s exact test. P-values <0.05 were considered statistically significant.

## Results

### Study population

The baseline characteristics of the study participants are summarized in **Table 1**. In total, 159 participants were recruited in this study among which, 99 were living with type 1 HIV and 60 were HIV-1 uninfected. The median age was higher in HIV-1 infected (P<0.0001) compared to uninfected group while participants in the non-infected group had a higher level of education than those in the infected group (P<0.0001). More women were living with HIV than men (P = 0.0012). The seroprevalence to HHV8 was 20.12% in this study and was not affected by HIV status (P >0.9999)

**Table 1:** General characteristics of the population.

Variables		All participants (N = 159)	HIV-1 uninfected (n = 60)	HIV-1 infected (n = 99)		P-values
Age (years) [Median (IQR)]		38 (28 – 45)	27.5 (24.25 – 34.5)	40 (35 – 48)		<0.0001
Sex	Male (%) Female (%)	47 (29.6) 112 (70.4)	27 (45) 33 (55)	20 (20.2) 79 (79.8)		0.0012
Education level [Median (IQR)]		2 (2-4)	4 (1-2)	2 (2-4)		<0.0001
HHV-8 Seropositivity (n/%)		32 (20.12)	12 (20)	20 (20.2)		>0.9999
				Undetectable Viral load (n = 44)	Detectable Viral loal (n = 55)	
CD4 LT count (cells/µL) [Median (IQR)]		-	-	566 (343.8 – 724.5)	184 (70 – 431)	<0.0001
CD4 LT count ≥ 500 (cells/µL) [n/%]		-	-	28 (63.64)	10 (18.18)	<0.0001
350 – 499 (cells/µL) CD4 LT count [n/%]		-	-	5 (11.36)	8 (14.55)	0.7684
200 – 349 (cells/µL) CD4 LT count [n/%]		-	-	7 (15.91)	9 (16.36)	>0.9999
CD4 LT count <200 (cells/µL) [n/%]		-	-	4 (9.09)	28 (50.91)	<0.0001
HHV-8 seropositivity (n/%)		-	-	4 (9.09)	16 (29.09)	0.0219
Duration of ARTs taking (month) [Median (IQR)]		-	NA	54 (22-132)	24 (1-84)	0.0028
ART taking (n/%)		-	NA	44 (100)	50 (90.91)	0.0638

IQR, Inter Quartile Range; HIV, Human Immunodeficiency Virus; ART, Antiretroviral Tritherapie: P values represent comparisons between HIV negative and HIV positive; and comparison in HIV positive group between undetectable and detectable viral load.

In the HIV-1 group, 44 (44,4%) had undetectable viral load and 55 (55,6%) had a detectable viral load. The median CD4 T cell count was significantly higher among the HIV-1-infected group with undetectable viral load compared to those with detectable viral load (P < 0.0001). The percentage of HIV-1-infected participants without immune suppression (CD4 T cell count ≥500 cells/µL) was significantly higher among those with an undetectable viral load, while the percentage of HIV-1-infected individuals with severe immune suppression (CD4 T cell count < 200 cells/µL) was significantly higher among those with detectable viral load (P < 0.0001 for the both). Concerning HHV8, its seroprevalence was higher among HIV-1 infected participants with detectable viral loads compared to those with undetectable viral loads (PVL< 40 cp/ml; P = 0.0219). The median length of duration on ART use was significantly higher among HIV-1 infected participants with an undetectable viral load (UVL< 40 cp/ml) compared to those with detectable viral load (P = 0.0028). A similar trend but not significant (p=0.0638) was observed with the percentage of patients who took ARTs drugs.

### Soluble biomarkers of SIA is Higher in PLWH

**Figure 2:**
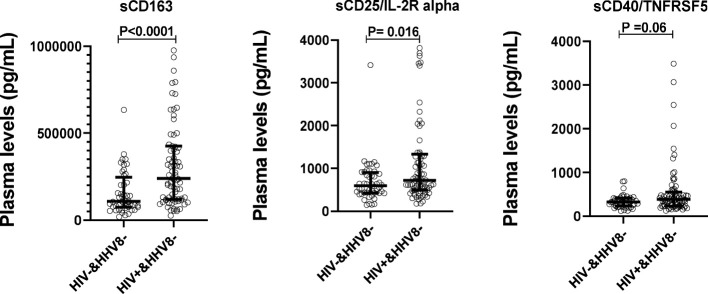
Plasma levels of solubles biomarkers of systemic immune activation stratified by HIV-1 status in adults. Plasma levels in sCD163, sCD25/IL-2R alpha, and sCD40/TNFRSF5 were compared between HIV+&HHV8- (N=79) and HIV-&HHV8- (N=48) adults using Mann- Whitney Rank Sum Test.

To evaluate the impact of HIV infection on the levels of sCD163, sCD25/IL-2R alpha, and sCD40/TNFRSF5, plasma levels of those biomarkers were compared between HIV-1 infected (HIV+) and HIV-1 uninfected adults (HIV-). Results from **Figure 2** showed that plasma levels of sCD163 and sCD25/IL-2R alpha were higher among adults living with HIV (ALWH) compared to HIV-1 uninfected adults (P < 0.0031 and P = 0.016 respectively). In addition, the Spearman rank correlations were used to explore associations between plasma levels of those biomarkers with CD4 T cell count, Viral load, and duration of ARTs taken in ALWH (**Table 2**). The results showed that plasma levels of sCD163 (r_s_ = - 0.2294, P = 0.0420), sCD25/IL-2R alpha (r_s_ = - 0.3580; P = 0.0012) and sCD40/TNFRSF5 (r_s_ = - 0.2978, P = 0.0077) correlated negatively with CD4 T cell count. Plasma levels of sCD163 (r_s_ = - 0.3704, P = 0.0013) and sCD25/IL-2Ralpha (r_s_ = - 0.2111, P = 0.0730) inversely correlated with the duration of ART use. Levels of sCD163 (r_s_ = 0.2387, P = 0.0342), sCD25/IL-2R alpha (r_s_ = 0.6280, P < 0.0001), and sCD40/TNFRSF5 (r_s_ = 0.4399, P < 0.0001) correlated with plasma viral load. These results suggest that HIV infection is associated with high levels of SIA.

**Table 2:** Correlation between plasma levels of solubles markers of systemic immune activation and CD4 LT count, viral load, and duration of ARTs taken in HIV-1 infected adults.

	LT CD4 count		Viral Load		Duration of ARVs taking	
sCD163	r_s_ = - 0.2294	P = 0.0420	r_s_ = 0.2387	P = 0.0342	r_s_ = - 0.3704	P = 0.0013
sCD25/IL-2R alpha	r_s_ = - 0.3580	P = 0.0012	r_s_ = 0.6280	P < 0.0001	r_s_ = - 0.2111	P = 0.0730
sCD40/TNFRSF5	r_s_ = - 0.2978	P = 0.0077	r_s_ = 0.4399	P < 0.0001	r_s_ = - 0.0369	P = 0.7563

The Sperman’s rank Order correlation test was used.

### Plasma levels of sCD163, sCD25/IL-2R alpha, sCD40/TNFRSF5, VEGF, and FGF-acidic are higher in donors with detectable viral loads (DVL)

**Figure 3:**
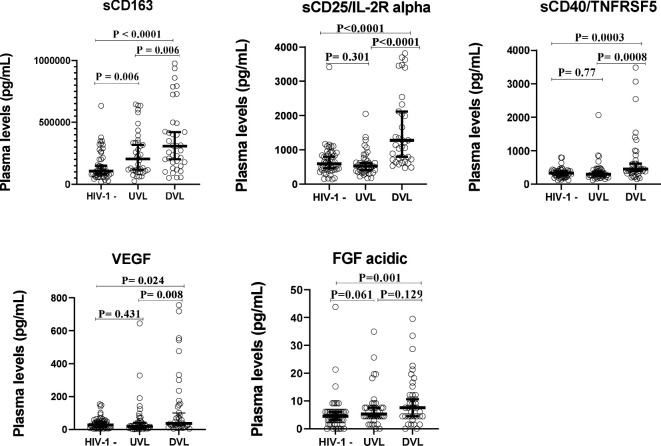
Plasma levels of solubles biomarkers of systemic immune activation and growth factors stratefied by Viral load in adults.

To determine whether the levels of soluble biomarkers of systemic immune activation (sCD163, sCD25/IL-2R alpha, sCD40/TNFRSF5) and that of growth factors (VEGF, FGF-acidic) varied with viral load in HIV mono-infected participants (HIV-1 positive/HHV-8 negative), plasma levels of these biomarkers were compared between ALWH with undetectable viral load (UVL), those with detectable viral load (DVL) and those uninfected to HIV-1. Results from **Figure 3** showed that plasma levels of sCD163 (P = 0.006), sCD25/IL-2R alpha (P < 0.0001), sCD40/TNFRSF5 (P = 0.0008), and VEGF (P = 0.008) were significantly higher among ALWH with DVL compared to those with UVL. Comparing plasma levels of soluble markers between ALWH co-infected with HHV-8 with a DVL (DVL & HHV-8 positive) to those mono-infected to HIV with a DVL (DVL & HHV-8 negative) showed no significant differences (**Supplementary Table 1**).

### Growth factors (VEGF and FGF acidic) levels correlated with 2 SIA biomarkers (sCD25/IL-2R alpha, and sCD40/TNFRSF5) in ALWH with UDL but not with sCD163

To investigate whether SIA in ALWH with UVL could drive the development of Kaposi sarcoma, Spearman rank correlations were used to explore associations between the growth factors and SIA in UVL and DVL donors as shown in **Table 3**. In ALWH with DVL the levels of VEGF and FGF acidic were positively and significantly correlated with the levels of all SIA biomarkers tested: sCD163 (r_s_ = 0.33, P= 0.038, and r_s_ = 0.48, P = 0.002); sCD25/IL-2R alpha (r_s_ =0.37, P = 0.018 and r_s_ = 0.57, P = 0.0001); sCD40/TNFRSF5 (r_s_ =0.55, P = 0.0002 and r_s_ = 0.68, P < 0.0001). Among ALWH with UVL, plasma levels of VEGF and FGF acidic were positively and significantly correlated with the levels of two SIA biomarkers tested: sCD25/IL-2R alpha (r_s_ = 0.39, P= 0.012 and r_s_ = 0.53, P = 0.0004) and sCD40/TNFRSF5 (r_s_ = 0.81, P< 0.0001 and r_s_ = 0.44, P = 0.0045).

**Table 3:** Correlation between plasma levels of growth factors and soluble biomarkers of SIA stratified by Viral load.

UVL			
	sCD163	sCD25/IL-2R alpha	sCD40/TNFRSF5
VEGF	r_s_ = 0.05 P = 0.74	r_s_ = 0.39 P = 0.012	r_s_ = 0.81 P < 0.0001
FGF acidic	r_s_ = 0.05 P = 0.72	r_s_ = 0.53 P = 0.0004	r_s_ = 0.44 P = 0.0045
DVL			
	sCD163	sCD25/IL-2R alpha	sCD40/TNFRSF5
VEGF	r_s_ = 0.33 P = 0.038	r_s_ = 0.37 P = 0.018	r_s_ = 0.55 P = 0.0002
FGF acidic	r_s_ = 0.48 P = 0.002	r_s_ = 0.57 P = 0.0001	r_s_ = 0.68 P < 0.0001

UVL, Undetectable Viral Load; DVL, Detectable Viral Load; VEGF, Vascular Endothelium Growth Factor; FGF-acidic, Fibroblast Growth Factor acidic.

The Spearman’s rank Order correlation test was used: r_s_ = coefficient of correlation; P = coefficient of significance.

### Plasma levels of sCD163, sCD25/IL-2R alpha, sCD40/TNFRSF5, VEGF and FGF-acidic are elevated in HIV-1 infected adults with seropositivity to HHV8

**Figure 4:**
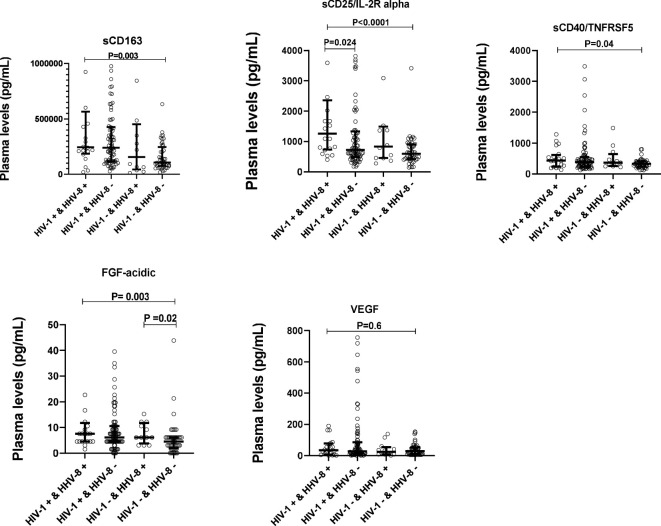
Plasma levels of solubles biomarkers of systemic immune activation and growth factors in HIV-1 and HHV8 in adults. Plasma levels of sCD163, sCD25/IL-2R alpha, sCD40/TNFSF5, FGF-acidic and VEGF were compared between HIV+&HHV8+ (N = 20) and HIV-&HHV8- (N = 48) adults using Mann-Whitney Rank Sum test.

To determine whether the levels of soluble biomarkers of systemic immune activation (sCD163, sCD25/IL-2R alpha, sCD40/TNFRSF5) and that of growth factors (VEGF, FGF-acidic) varied with HIV-1 and HHV8 co-infection, plasma levels of these biomarkers were compared between ALWH with seropositivity to HHV8 (HIV+&HHV8+) and those uninfected to HIV-1 without seropositivity to HHV8 (HIV-&HHV8-); those uninfected to HIV-1 with seropositivity to HHV8 (HIV-&HHV8+) and those infected to HIV-1 without seropositivity to HHV8 (HIV+&HHV8-). As can be seen in **Figure 4** and **Table 4** plasma levels of sCD163 (P = 0.003), sCD25/IL-2R alpha (P < 0.0001), sCD40/TNFRSF5 (P = 0.041), and FGF acidic (P = 0.003) were significantly higher among HIV+&HHV8+ compared to double negative HIV-&HHV8-. In addition, plasma levels of sCD25/IL-2R alpha were significantly higher among HIV+&HHV8+ compared to HIV+/HHV8- (P = 0.024). A similar trend was observed when comparing plasma levels of FGF acidic between HIV- and HHV8+ mono-infected and HIV- and HHV8- uninfected (P = 0.023) groups. No significant difference has been observed between HIV+&HHV8+ and HIV-&HHV8+ for sCD163 (P = 0.307); sCD25/IL-2R alpha (P = 0.157) and sCD40/TNFRSF5 (P = 0.7445) and between HIV+ and HHV8+ co-infection and HIV+ and HHV8- mono-infection for sCD163 (P = 0.520); sCD40/TNFRSF5 (P = 0.529).

**Table 4:** Median levels of markers of SIA and angiogenesis in HIV-1 and HHV-8.

	HIV+&HHV8+	HIV-&HHV8-	P	HIV+&HHV8-	HIV-&HHV8-	P	HIV-&HHV8+	HIV-&HHV8-	P
**sCD163**	244612 (186310 - 565526)	107643 (73805 -246830)	0.0031	240588 (118952 -426568)	107643 (73805 -246830)	<0.0001	156672 (43183 - 452162)	107643 (73805 -246830)	0.8165
**sCD25/IL-2R alpha**	1262 (730.2 - 2354)	595.0 (422.8 - 907.4)	<0.0001	722.1 (487.4 - 1333)	595.0 (422.8 - 907.4)	0.0160	834.2 (457.1 - 1487)	595.0 (422.8 - 907.4)	0.1345
**sCD40/TNFRSF5**	438.9 (235.6 - 622.9)	327.1 (232.5 - 411.1)	0.0405	383.8 (236.3 - 550.5)	327.1 (232.5 - 411.1)	0.0602	368.4 (257.3 - 649.0)	327.1 (232.5 - 411.1)	0.2428
**VEGF**	34.33 (6.39 - 78.63)	28.5 (10.68 - 52.42)	0.6912	27.67 (10.77 - 86.24)	28.5 (10.68 - 52.42)	0.4056	23.4 (8.077 - 55.08)	28.5 (10.68 - 52.42)	0.6060
**FGF-acidic**	7.558 (4.535 - 11.77)	4.535 (1.901 - 6.984)	0.0035	6.084 (4.535 - 10.58)	4.535 (1.901 - 6.984)	0.0035	6.084 (3.802 - 11.77)	4.535 (1.901 - 6.984)	0.0230

HIV+&HHV8+, positive to HIV-1 and seropositive to HHV8; HIV+&HHV8-, positive to HIV-1 and seronegative to HHV8; HIV-&HHV8+, negative to HIV-1 and seropositive to HHV8; HIV-&HHV8-, negative to HIV-1 and seronegative to HHV8. Values outside the brackets correspond to the median while values in brackets correspond to the inter-quartile range (25 and 75 percentiles).

### High plasma levels of VEGF and FGF acidic correlated with sCD163, sCD25/IL-2R alpha, and sCD40/TNFRSF5 levels in HIV-1 and HHV8 co-infected adults

Spearman rank correlations were used to explore associations between plasma levels of growth factors tested (VEGF, FGF-acidic) and soluble biomarkers of systemic immune activation (sCD163, sCD25/IL-2R alpha, sCD40/TNFRSF5) in HIV+&HHV8+, HIV+&HHV8- and HIV-&HHV8+ adults (**Table 5**). Results showed that plasma levels of VEGF and FGF acidic were positively and significantly correlated with the levels of sCD163 (r_s_ = 0.58, P = 0.0067 and r_s_ = 0.43, P = 0.058); sCD40/TNFRSF5 (r_s_ = 0.59, P = 0.0064 and r_s_ = 0.51, P = 0.022). although positive, this was not significant with sCD25/IL-2R alpha (r_s_ = 0.31, P = 0.17 and r_s_ = 0.37, P = 0.102) in HIV+/HHV8+ adults. Similar trends were observed in the levels of VEGF and FGF acidic with sCD163 (r_s_ = 0.25, P = 0.03 and r_s_ = 0.30, P = 0.006), sCD25/IL-2R alpha (r_s_ = 0.5, P<0.0001 and r_s_ = 0.55, P < 0.0001), sCD40/TNFRSF5 (r_s_ = 0.7, P<0.0001 and r_s_ = 0.59, P < 0.0001) among HIV+&HHV8- adults. Also, FGF acidic strongly correlated with sCD40/TNFRSF5 (r_s_ = 0.65 P = 0.025) but not with sCD163 and sCD25/IL-2R alpha in HIV-&HHV8+. Although positive, no significant correlation has been observed between VEGF and sCD163, sCD40/TNFRSF5, sCD25/IL-2R alpha in HIV-&HHV8+ adults (r_s_ = 0.25, P = 0.430; r_s_ = 0.41, P = 0.184; and r_s_ = 0.13, P = 0.696 respectively).

**Table 5:** Correlation between plasma levels of growth factors and soluble biomarkers of systemic immune activation in relation to HIV-1 and HHV8 status.

HIV+&HHV8+			
	sCD163	sCD25/IL-2R alpha	sCD40/TNFRSF5
**VEGF**	r_s_ = 0.58 P = 0.006	r_s_ = 0.31 P = 0.177	r_s_ = 0.59 P = 0.006
**FGF acidic**	r_s_ = 0.43 P = 0.058	r_s_ = 0.37 P = 0.102	r_s_ = 0.51 P = 0.022
HIV+&HHV8-			
	sCD163	sCD25/IL-2R alpha	sCD40/TNFRSF5
**VEGF**	r_s_ = 0.24 P = 0.03	r_s_ = 0.50 P < 0.0001	r_s_ = 0.71 P < 0.0001
**FGF acidic**	r_s_ = 0.30 P = 0.006	r_s_ = 0.55 P < 0.0001	r_s_ = 0.59 P < 0.0001
HIV-&HHV8+			
	sCD163	sCD25/IL-2R alpha	sCD40/TNFRSF5
**VEGF**	r_s_ = 0.25 P = 0.430	r_s_ = 0.13 P = 0.696	r_s_ = 0.41 P = 0.184
**FGF acidic**	r_s_ = 0.46 P = 0.137	r_s_ = 0.49 P = 0.106	r_s_ = 0.65 P = 0.025

HIV+&HHV8+, positive to HIV-1 and seropositive to HHV8; HIV+&HHV8-, positive to HIV-1 and seronegative to HHV8; HIV-&HHV8+, negative to HIV-1 and seropositive to HHV8; HIV-&HHV8-, negative to HIV-1 and seronegative to HHV8; VEGF, Vascular Endothelium Growth Factor; FGF-acidic, Fibroblast Growth Factor acidic.

The Spearman’s rank Order correlation test was used: r_s_ = coefficient of correlation; P = coefficient of significance.

## Discussion

Kaposi’s sarcoma (KS) is the most common neoplasm that arises in HIV-infected patients. A better understanding of the relationship between HIV-1-associated immune activation and biomarkers of angiogenesis involve in the pathogenesis of KS is crucial for the identification of potential immunopathogenesis mechanisms linked to the risk of developing KS in HHV-8 seropositive adults. To date, the impact of HIV-1-associated systemic immune activation on the plasma level of some growth factors known as biomarkers of KS progression has not been extensively studied. This study aimed to investigate the relationship between soluble markers of HIV-1-related immune activation and the level of growth factors in HHV8 seropositive adults about KS pathogenesis. The seroprevalence of HHV-8 was 20.12% in the present study and was not affected by HIV status. This result is in accord with those from other studies in sub-Saharan Africa (27–29) and can be explained by the geographical variation associated with this oncogenic virus. We found that plasma concentrations of soluble biomarkers sCD163 and sCD25/IL-2R alpha were higher among HIV-1 infected compared to HIV-1 uninfected adults. In addition, plasma concentrations of all these soluble biomarkers of immune activation correlated negatively with LT CD4 count but positively with viral load. This is consistent with findings that suggest plasma levels of sCD163, a monocyte/macrophage activation biomarker, are elevated in HIV-infected individuals. Even under treatment with ART, abnormal immune activation persists (30–32). In addition, it has been demonstrated that HIV gene products, such as gp120 and Nef, directly stimulate the activation of lymphocytes and macrophages, resulting in the secretion of proinflammatory cytokines and chemokines (33). This study, is among the pioneers that show elevated plasma levels of sCD25/IL-2R alpha and sCD40/TNFRSF5 in PLWH compared to HIV-uninfected adults. We also show that sCD25/IL-2R alpha and sCD40/TNFRSF5 levels correlate positively with viral load but negatively with CD4 LT count. These data indicate that HIV infection is associated with a greater degree of immune activation. Although ART helps reduce inflammation it remains higher than in HIV-1-negative individuals Systemic immune activation has emerged as an essential component of the immunopathogenesis of HIV, it leads to faster disease progression and an accelerated decline of several immune competencies. It is a strong predictor of the development of comorbidity and even mortality in PLWH. This remains true but to a lesser degree in patients who are virally suppressed (34). We demonstrated in this study that in ALWH with UVL, two soluble biomarkers of SIA (sCD25/IL-2R alpha and sCD40/TNFRSF5) were positively associated with plasma levels of growth factors (VEGF and FGF acidic) implicated in the pathogenesis of Kaposi sarcoma. This further highlights the role of SIA in the pathogenesis of KS and shows an increased risk in HIV patients even when they are virally suppressed. We found no difference between SIA and angiogenesis markers in DVL/HHV8 + vs DVL/HHV8-, and, in UVL/HHV8+ vs UVL/HHV8- participants. These suggest that soluble markers of SIA and angiogenesis are not influenced by the infection with HHV-8. However, our lack of statistical power in the DVL/HHV8 + and UVL/HHV8+ pushes us to ascertain these findings in subsequent studies.

Our results reveal that co-infection to HIV-1 and HHV-8 is associated with increased immune activation, as reflected by higher levels of sCD163, sCD25/IL-2R alpha, and sCD40/TNFRSF5 and angiogenesis as assessed by the high levels of FGF acidic, also we find high level of FGF acidic in HHV-8 mono-infected adults. These results suggest that persons with co-infection have higher levels of systemic immune activation markers and may be implicated in the development of KS, and FGF acidic showed potential to be a biomarker of HHV-8 infection in HIV-uninfected adults. Given the evidence that HHV-8 is not sufficient for the development of KS, there is a great need to identify additional factors and mechanisms that may be implicated in the pathogenesis of KS in ALWH. It is worth noting that elevated levels of soluble biomarkers are associated with the severity of other diseases. Several studies have shown elevated sCD163 in the serum of tumor patients and that increased levels were linked to poor prognosis in several cancers (35–38). About sCD25/IL-2R alpha, the levels of this biomarker have been observed to increase in hepatocellular carcinoma and were considered a mediator of T-cell suppression and tumor progression (39, 40). Sebastian and colleagues (41) demonstrated that a high circulating level of sCD40/TNFRSF5 is a marker of liver metastasis risk in rectal cancer. Although the roles of sCD163, sCD25/IL-2R alpha, and sCD40/TNFRSF5 in the control of other diseases have been proven, to our knowledge, this study is the first to demonstrate the association of HIV-1 and HHV-8 coinfection with high levels of these three systemic immune activation biomarkers tested.

The biomarkers of angiogenesis and progression in our study reveal new findings and suggest that HIV-1-related systemic immune activation is associated with high VEGF and FGF acidic levels in HHV8 seropositive adults. In fact, VEGF, an angiogenic and vascular permeability factor, has been postulated as a major angiogenic and growth factor in KS and some previous data indicate that VEGF in synergy with basic FGF induces endothelial cell growth and angiogenesis (25–27). In addition, Felipe and colleagues (42) reported that KS lesions and KS-derived spindle cell cultures co-express high levels of basic FGF and VEGF, which is promoted by the inflammatory cytokines such as tumor necrosis factor (TNF), interleukin-1(IL-1), and interferon-γ (IFN-γ) in PLWH. Although high levels of VEGF and basic FGF have previously been associated with pro-inflammatory cytokines, this study demonstrated for the first time that high levels of VEGF and acidic FGF are associated with soluble biomarkers of systemic immune activation, namely soluble CD163, soluble CD 25/Inerleukine-2R alpha and soluble CD40/Tumor necrosis factor receptor-SF5 in HIV-1 and HHV8 co-infected adults

The limitation of this study is that it was a cross-sectional study, more data on systemic immune activation biomarkers at different time points are necessary to make a good conclusion on the implication of HIV-1- associated immune activation in the risk of developing KS among HHV-8 seropositive adults. Also, the lack of HHV8 viral load data in this study limited the evaluation of the viral activity of HHV8. The data of this study will serve as preliminary data, in-depth study needs to be conducted for the identification of biomarkers linked to the risk of developing Kaposi sarcoma among people co-infected with HIV-1 and HHV8.

## Conclusion

In HIV-1 infection, soluble biomarkers of systemic immune activation are associated with high levels of biomarkers of angiogenesis involves in the pathogenesis of KS in HHV-8 seropositive adults. This may contribute to an increase in the risk among HIV-1 and HHV-8 coinfected adults to develop KS even under controlled viral loads.

## Data availability statement

The original contributions presented in the study are included in the article/**Supplementary Material**. Further inquiries can be directed to the corresponding author.

## Ethics statement

This study was reviewed and approved by Professor Lazare Kaptué, President of the National Ethics Committee of Cameroon (Ethical Clearance 2019/07/N°38/CE/CNERSH/SP) and by Dr Casimir Doho Beye, Chair Person of the Regional Ethics Committee, Centre Region (Ethical Clearance No CE2023/CRERSHC/2022) for Human Health Research. The studies were conducted in accordance with the local legislation and institutional requirements. The participants provided their written informed consent to participate in this study.

## Author contributions

BN and RM conceived and designed the study. BN, DA, TS, HA, and GE help with data acquisition. BN, CN, DA, TS, and RS performed most of the experiments. BN, LE, MB, and CN analysed and interpreted the data. BN and LE drafted the manuscript. RM, LN, SO, MB, and KO substantively reviewed the final manuscript for important intellectual content. RM, LN, and RL supervised the study. All authors contributed to the article and approved the submitted version.

## References

[1] Haverkos WH. Multifactorial etiology of Kaposi’s sarcoma: a hypothesis. J Biosci (2008) 33:643–51. doi: 10.1007/s12038-008-0084-x 19179752

[2] Antman K Chang Y. Kaposi’s sarcoma. New Engl J Med (2000) 342:1027–38. doi: 10.1056/NEJM200004063421407 10749966

[3] Phipps W Ssewankambo F Nguyen H Saracino M Wald A Corey L. Gender differences in clinical presentation and outcomes of epidemic Kaposi sarcoma in Uganda. PloS One (2010) 5:e13936. doi: 10.1371/journal.pone.0013936 21103057 PMC2980479

[4] Gallafent JH Buskin SE de Turk PB Aboulafia DM. Profile of patients with Kaposi’s sarcoma in the era of highly active antiretroviral therapy. J Clin Oncol (2005) 23:1253–60. doi: 10.1200/JCO.2005.04.156 15718323

[5] Hleyhel M Belot A Bouvier AM Tattevin P Pacanowski J Genet P. Risk of AIDS-defining cancers among HIV-1-infected patients in France between 1992 and 2009: results from the FHDHANRS CO4 cohort. Clin Infect Dis (2013) 57:1638–47. doi: 10.1093/cid/cit497 23899679

[6] Uldrick TS Whitby D. Update on KSHV epidemiology, Kaposi sarcoma pathogenesis, and treatment of Kaposi sarcoma. Cancer Lett (2011) 305:150–62. doi: 10.1016/j.canlet.2011.02.006 PMC3085592 21377267

[7] Younas M Psomas C Reynes C Cezar R Kundura L Portalès P. Residual viremia is linked to a specific immune activation profile in HIV-1-infected adults under efficient antiretroviral therapy. Front Immunol (2021) 12:663843. doi: 10.3389/fimmu.2021.663843 33859653 PMC8042152

[8] Lederman MM Funderburg NT Sekaly RP Klatt NR Hunt PW. Residual immune dysregulation syndrome in treated HIV infection. Adv Immunol (2013) 119:51–83. doi: 10.1016/B978-0-12-407707-2.00002-3 23886064 PMC4126613

[9] Neuhaus J Jacobs DR Jr Baker JV Calmy A Duprez D La Rosa A. Markers of inflammation, coagulation, and renal function are elevated in adults with HIV infection. J Infect Dis (2010) 201:1788–95. doi: 10.1086/652749 PMC2872049 20446848

[10] Lederman MM Calabrese L Funderburg NT Clagett B Medvik K Bonilla H. Immunologic failure despite suppressive antiretroviral therapy is related to activation and turnover of memory CD4 cells. J Infect Dis (2011) 204:1217–26. doi: 10.1093/infdis/jir507 PMC3218674 21917895

[11] Sandler NG Wand H Roque A Law M Nason MC Nixon DE. Plasma levels of soluble CD14 independently predict mortality in HIV infection. J Infect Dis (2011) 203:780–90. doi: 10.1093/infdis/jiq118 PMC3071127 21252259

[12] Jong E Louw S van Gorp EC Meijers JC ten Cate H Jacobson BF. The effect of initiating combined antiretroviral therapy on endothelial cell activation and coagulation markers in South African HIV-infected individuals. Thromb Haemost. (2010) 104:1228–34. doi: 10.1160/TH10-04-0233 20886182

[13] Hunt PW Martin JN Sinclair E Bredt B Hagos E Lampiris H. T cell activation is associated with lower CD4 + T cell gains in human immunodeficiency virus–infected patients with sustained viral suppression during antiretroviral thera$$ppy. J Infect Dis (2003) 187:1534–43. doi: 10.1086/374786 12721933

[14] Burdo TH Lo J Abbara S Wei J DeLelys ME Preffer F. Soluble CD163, a novel marker of activated macrophages, is elevated and associated with noncalcified coronary plaque in HIV-infected patients. J Infect Dis (2011) 204:1227–36. doi: 10.1093/infdis/jir520 PMC3203384 21917896

[15] Burdo TH Lentz MR Autissier P Krishnan A Halpern E Letendre S. Soluble CD163 made by monocyte/macrophages is a novel marker of HIV activity in early and chronic infection prior to and after anti-retroviral therapy. J Infect Dis (2011) 204:154–63. doi: 10.1093/infdis/jir214 PMC3105035 21628670

[16] Zhi Y Gao P Xin X Li W Ji L Zhang L. Clinical significance of sCD163 and its possible role in asthma (Review). Mol Med Rep (2017) 15:2931–9. doi: 10.3892/mmr.2017.6393 PMC5428902 28350095

[17] Rittig N Svart M Jessen N Møller N Møller HJ Grønbæk H. Macrophage activation marker sCD163 correlates with accelerated lipolysis following LPS exposure: a human-randomized clinical trial. Endocr Connect. (2018) 1:107–14. doi: 10.1530/EC-17-0296 PMC5754508 29295869

[18] Contin C Pitard V Delmas Y Pelletier N DeFrance T Moreau JF. Potential role of soluble CD40 in the humoral immune response impairment of uraemic patients. Immunology (2003) 110:131–40. doi: 10.1046/j.1365-2567.2003.01716.x PMC1783029 12941150

[19] Hock BD McKenzie JL Patton NW Drayson M Taylor K Wakeman C. Circulating levels and clinical significance of soluble CD40 in patients with hematologic Malignancies. Cancer (2006) 106:21482157. doi: 10.1002/cncr.21816 16598754

[20] Schmilovitz-Weiss H Belinki A Pappo O Sulkes J Melzer E Kaganovski E. Role of circulating soluble CD40 as an apoptotic marker in liver disease. Apoptosis (2004) 9:205–10. doi: 10.1023/B:APPT.0000018802.95600.25 15004517

[21] Ravaggi1 A Gambino A Ferrari F Olivari1 A Zanotti L Romani C. VEGF-D serum level as a potential predictor of lymph node metastasis and prognosis in vulvar squamous cell carcinoma patients. Front Oncol (2022) 12:818613. doi: 10.3389/fonc.2022.818613 35463308 PMC9026339

[22] Cornali E Zietz C Benelli R Weninger W Masiello L Breier G. Vascular endothelial growth factor regulates angiogenesis and vascular permeability in Kaposi’s sarcoma. Am J Pathol (1996) 149:1851–69. PMC1865351 8952523

[23] Masood R Cai J Zheng T Smith DL Naidu Y Gill PS. Vascular endothelial growth factor/vascular permeability factor is an autocrine growth factor for AIDS-Kaposi sarcoma. Proc Natl Acad Sci USA (1997) 94:979–84. doi: 10.1073/pnas.94.3.979 PMC19625 9023368

[24] Nakamura S Murakami-Mori K Rao N Weich HA Rajeev B. Vascular endothelial growth factor is a potent angiogenic factor in AIDS-associated Kaposi’s sarcoma-derived spindle cells. J Immunol (1997) 158:4992–5001. doi: 10.4049/jimmunol.158.10.4992 9144519

[25] Long-Term HIV Treatment adherence for kew populations: 18 mai 2023- Ministry of Public Health Cameroon. Guidelines for the implemantation of Test and Treat strategy in Cameroon. Yaoundé, Cameroon (2017) 142. Available at: https://www.differentiatedservicedelivery-org/wp-content/uploads/cameroon.pdf.

[26] Livo FE Honore A Dieudonne N Michael B Idriss T Celine NN. Expression profiles of miR3181 and miR199a in plasma and placenta of virally suppressed HIV-1 infected Cameroonian pregnant women at delivery. PloS One (2022) 17(5):e0268820. doi: 10.1371/journal.pone.0268820 35594307 PMC9122233

[27] Newton R Ziegler J Bourboulia D Casabonne D Beral V Mbidde E. The sero-epidemiology of Kaposi’s sarcoma-associated herpesvirus (KSHV/HHV-8) in adults with cancer in Uganda. Int J Cancer (2003) 103:226–32. doi: 10.1002/ijc.10817 12455037

[28] Malope BI Pfeiffer RM Mbisa G Stein L Ratshikhopha EM O’Connell DL. Transmission of Kaposi sarcoma-associated herpesvirus between mothers and children in a South African population. J Acquir Immune Defic Syndr (2007) 44:351–5. doi: 10.1097/QAI.0b013e31802f12ea 17195763

[29] Dollard SC Butler LM Jones AM Mermin JH Chidzonga M Chipato T. Substantial regional differences in human herpesvirus 8 seroprevalence in sub-saharan africa: insights on the origin of the “KS belt”. Int J Cancer (2010) 127(10):2395–401. doi: 10.1002/ijc.25235 PMC2895015 20143397

[30] Knudsen AD Bouazzi R Afzal S Gelpi M Benfield T Høgh J. Monocyte count and soluble markers of monocyte activation in people living with HIV and uninfected controls. BMC Infect Dis (2022) 22:451. doi: 10.1186/s12879-022-07450-y 35546661 PMC9097376

[31] McKibben RA Margolick JB Grinspoon S Li X Palella FJ Kingsley LA. Elevated levels of monocyte activation markers are associated with subclinical atherosclerosis in men with and those without HIV infection. J Infect Dis (2015) 211:1219–28. doi: 10.1093/infdis/jiu594 PMC4402336 25362192

[32] O’Halloran JA Dunne E Gurwith M Lambert JS Sheehan GJ Feeney ER. The effect of initiation of antiretroviral therapy on monocyte, endothelial, and platelet function in HIV-1 infection. HIV Med (2015) 16:608–19. doi: 10.1111/hiv.12270 26111187

[33] Appay V Sauce D. “Immune activation and inflammation in HIV-1 infection: causes and consequences”. J Pathol (2008) 214:231–41. doi: 10.1002/path.2276 18161758

[34] Miller CJ Baker JV Bormann AM Erlandson KM Hullsiek KH Justice AC. Adjudicated morbidity and mortality outcomes by age among individuals with HIV infection on suppressive antiretroviral therapy. PloS One (2014) 9:e95061. doi: 10.1371/journal.pone.0095061 24728071 PMC3984283

[35] Kazankov K Rode A Simonsen K Villadsen GE Nicoll A Møller HJ. Macrophage activation marker soluble CD163 may predict disease progression in hepatocellular carcinoma. Scand J Clin Lab Invest. (2016) 76:64–73. doi: 10.3109/00365513.2015.1099722 26549495

[36] No JH Moon JM Kim K Kim YB. Prognostic significance of serum soluble CD163 level in patients with epithelial ovarian cancer. Gynecol Obstet Invest. (2013) 75:263–7. doi: 10.1159/000349892 23595052

[37] Nederby L Roug AS Knudsen SS Skovbo A Kjeldsen E Moller HJ. Soluble CD163 as a prognostic biomarker in B-cell chronic lymphocytic leukemia. Leuk Lymphoma. (2015) 56:11. doi: 10.3109/10428194.2015.1026899 25747973

[38] Andersen MN Abildgaard N Maniecki MB Møller HJ Andersen NF. Monocyte/macrophage-derived soluble CD163: a novel biomarker in multiple myeloma. Eur J Haematol (2014) 93:1. doi: 10.1111/ejh.12296 24612259

[39] Cabrera R Ararat M Cao M Xu Y Wasserfall C Atkinson MA. Hepatocellular carcinoma immunopathogenesis: clinical evidence for global T cell defects and an immunomodulatory role for soluble CD25 (SCD25). Dig. Dis Sci (2010) 55:484–95. doi: 10.1007/s10620-009-0955-5 PMC3161029 19714465

[40] Cabrera R Ararat M Eksioglu EA Cao M Xu Y Wasserfall C. Influence of serum and soluble CD25 (SCD25) on regulatory and effector T-cell function in hepatocellular carcinoma. Scand J Immunol (2010) 72:293–301. doi: 10.1111/j.1365-3083.2010.02427.x 20883314 PMC2951624

[41] Sebastian M Annette T Hanna A Arne Mide S Kjersti F Svein D. The circulating soluble form of the CD40 costimulatory immune checkpoint receptor and liver metastasis risk in rectal cancer. Br J Cancer. (2021) 125:240–6. doi: 10.1038/s41416-021-01377-y PMC8292313 33837301

[42] Felipe S Phillip DM Rita G Yoshiki W Vivien K Kimberly K. Endothelial growth factor and basic fibroblast growth factor present in kaposi’s sarcoma (KS) are induced by inflammatory cytokines and synergize to promote vascular permeability and KS lesion development. Am J Pathol (1998) 152:1433–43. PMC1858461 9626048

