# Supplementary material for: Soluble biomarkers of HIV-1-related systemic immune activation are associated with high plasma levels of growth factors implicated in the pathogenesis of Kaposi sarcoma in adults
Source: Front Immunol. 2023 Sep 18;14:1216480. doi: 10.3389/fimmu.2023.1216480 (PMC10552755; doi:10.3389/fimmu.2023.1216480)
Supplement: Supplementary file 1 [file Table_1.docx]

Supplementary Table 1: Median levels of soluble markers of SIA and angiogenesis in HIV-1 and HHV-8 stratified by Viral Load

|  | **UVL & HHV-8 negative (N = 40)** | **UVL & HHV-8 positive (N = 4)** | **P-values** | **DVL &HHV-8 negative (N = 39)** | **DVL & HHV-8 positive (N = 16)** | **P-valus** |
| --- | --- | --- | --- | --- | --- | --- |
| **sCD163** | 205672 (109760 - 352890) | 176945 (82603 - 234103) | 0,5174 | 309678 (177534 -500628) | 309814 (207775 - 843620) | 0,5631 |
| **sCD25/IL-2Rα** | 531,2 (366,0 - 711,3) | 678,9 (434,8 - 1029) | 0,4389 | 1278 (724,1 - 3402) | 1601 (778,1 - 3309) | 0,5385 |
| **sCD40/TNFR2SF5** | 297,0 (226,7 - 435,4) | 304,5 (188,3 - 421,7) | 0,6462 | 450,8 (335,3 - 868,7) | 495,9 (278,0 - 860,5) | 0,8761 |
| **VEGF** | 19,22 (7,816 - 50,07) | 27,25 (1,937 - 155,1) | 0,8358 | 36,89 (17,30 - 157,0) | 34,33 (13,10 - 78,63) | 0,3137 |
| **FGF acidic** | 5,309 (4,535 - 3,415) | 4,535 (7,558 - 124,7) | 0,8482 | 7,558 (4,535 - 12,17) | 7,558 (4,535 - 11,77) | 0,8136 |

UVL: Undetectable Viral Load; DVL: Detectable Viral Load; VEGF: Vascular Endothelium Growth Factor ; FGF-acidic: Fibroblast Growth Factor acidic

Values outside the brackets correspond to the median while values in brackets correspond to the inter-quartile range (25 and 75 percentiles)
